# What are the determinants of older people adopting communicative e-health services: a meta-ethnography

**DOI:** 10.1186/s12913-023-10372-3

**Published:** 2024-01-11

**Authors:** Ayse Aslan, Freda Mold, Harm van Marwijk, Jo Armes

**Affiliations:** 1 School of Health Sciences, University of Surrey, Guildford, UK; 2 Brighton and Sussex Medical School, Brighton, UK

**Keywords:** Aged, Telemedicine, Digital Technology, Information Technology, Social Support, Qualitative Research

## Abstract

**Background:**

Gradually, society has shifted more services online, with COVID-19 highlighting digital inequalities in access to services such as healthcare. Older adults can experience such digital inequalities, yet this group is also more likely to need medical appointments, compared to younger people. With the growing digitalisation of healthcare, it is increasingly important to understand how older people can best use communicative e-health services to interact with healthcare services. This is especially if older adults are to access, and actively interact with health professionals/clinicians due to their general health decline. This review aims to synthesise older adults’ experiences and perceptions of communicative e-health services and, in turn, identify barriers and facilitators to using communicative e-health services.

**Methods:**

A meta-ethnography was conducted to qualitatively synthesise literature on older adults’ experiences of using communicative e-health services. A systematic search, with terms relating to ‘older adults’, ‘e-health’, ‘technology’, and ‘communication’, was conducted on six international databases between January 2014 and May 2022. The search yielded a total of 10 empirical studies for synthesis.

**Results:**

The synthesis resulted in 10 themes that may impact older adults’ perceptions and/or experiences of using communicative e-health services. These were: 1) health barriers, 2) support networks, 3) application interface/design, 4) digital literacy, 5) lack of awareness, 6) online security, 7) access to digital devices and the internet, 8) relationship with healthcare provider(s), 9) in-person preference and 10) convenience. These themes interlink with each other.

**Conclusion:**

The findings suggest older adults’ experiences and perceptions of communicative e-health services are generally negative, with many reporting various barriers to engaging with online services. However, many of these negative experiences are related to limited support networks and low digital literacy, along with complicated application interfaces. This supports previous literature identifying barriers and facilitators in which older adults experience general technology adoption and suggests a greater emphasis is needed on providing support networks to increase the adoption and usage of communicative e-health services.

**Supplementary Information:**

The online version contains supplementary material available at 10.1186/s12913-023-10372-3.

## Background

Society has shifted more towards the digital world since the COVID-19 pandemic, with a specific increase in the delivery of virtual health services [1]. E-health service refers to the use of information and communication technologies to promote access to quality healthcare services, such as electronic health records, mHealth, telemedicine, web-based health services, and clinical decision support systems [2]. E-health services have the potential to benefit older adults and the wider population, but only when the individual has some degree of e-health literacy [3]. However, many older adults may not be able to keep up with these developments, which could add to their social isolation and feelings of loneliness. The introduction of digital services can exacerbate inequalities [4]. Clinicians report an increase in video consultations, however, a systematic review found that uptake and utilisation were more prevalent amongst younger and employed adults [5]; likewise, Schifelding et al. [6] found older people tended to use them less. This supports the notion of digital inequalities within the healthcare sector, with many older people potentially missing out on care. Whilst E-health services help to strengthen patient-centred care [7], the mechanisms by which ageing impacts communicative e-health service adoption are not currently well understood. However, more research is required regarding older adult's willingness to use digital technologies in health care.

Over the years, there has been a growing body of literature focusing on e-health services and older adults [8–10]. Several common barriers and facilitators to e-health services have been identified, which include but are not limited to, poorly designed interface [11–13], high cost of technology [14], privacy and security issues [8, 13, 15, 16], chronic illness [17–19] and lack of digital skills [9, 16, 19–21]. Facilitators of engaging with e-health services for this group comprises of support from family and healthcare providers [9, 22–25], doctors' recommendations [26], access to technology and the internet [10, 27], and high levels of education [10, 26, 28].

It is imperative to conduct a synthesis of older adults’ experiences and perceptions of communicative e-health services to better understand how they can utilise these in everyday life. This is due to the digital inequalities older adults are more likely to experience, paired with the rapid increase in e-health services since the COVID-19 pandemic. We define communicative e-health services as any online health service a patient receives or seeks out, that involves them actively interacting with a healthcare provider. This review builds upon research from Kapadia et al.’s [21] systematic review which identified key issues impacting information and communication technologies (ICT) adoption amongst older adults. Kapadia et al. incorporated older adults’ perspectives in their review, along with the views of healthcare professionals and management. Since Kapadia et al.’s review, there have been several reviews on older adults and e-health services, however, these are either quantitative reviews or qualitative synthesises which did not incorporate direct perceptions and/or experiences of older adults. To the authors’ knowledge, no review has been conducted of qualitative literature that focuses on older adults’ experiences and perceptions of e-health services, and more specifically, the communication aspect of e-health services. This review will provide overarching explanations derived from qualitative studies exploring the personal experiences and perceptions of older adults to increase our understanding of older adults’ interaction with communicative e-health services, and the barriers and facilitators of use.

As there is little known about how ageing impacts technology adoption, this review aims to provide overarching explanations derived directly from the personal experiences and opinions of older adults, extracted from qualitative studies. These explanations will increase our understanding of older adults’ interaction within communicative e-health services. Moreover, communicative e-health services are the focus of this meta-ethnography due to the recent increase in online communication as a consequence of COVID-19, which has impacted face-to-face health services. Therefore, as older adults are more likely to use healthcare services, along with communicative e-health services continuing to be used, it is important to understand older people’s experiences and opinions of communicative e-health services and to identify, any barriers, and facilitators. This knowledge can then be used by healthcare providers, to increase older adults’ adoption and access to communicative e-health services.

### Aims of the study

This meta-ethnography aims to synthesise qualitative studies exploring older adults’ perceptions and/or experiences of using communicative e-health services, which will help guide healthcare providers in improving their communicative e-health services, making them more age-friendly.

The objectives are to identify:

1. older adults’ experiences of communicative e-health services
2. barriers and facilitators to older adults adopting communicative e-health services

## Methods

### Review design

Noblit and Hare’s [29] seven-step approach to meta-ethnography was utilised for this review. Meta-ethnography is widely used within health research [30–32] when synthesising qualitative studies. It explores the phenomenon of interest as the meta-ethnographic synthesis translates the primary qualitative studies into one another. By doing this, it aims to generate new understandings of the phenomenon. Meta-ethnographic synthesis aims to interpret the findings of the literature, rather than provide an aggregated summary which other qualitative syntheses do. This is done by preserving key concepts from the original text while making new interpretations of the studies.

### Search strategy/study identification

The review was registered with PROSPERO on 25^th^ March 2022 (registration number: CRD42022320440).

**Fig. 1:**
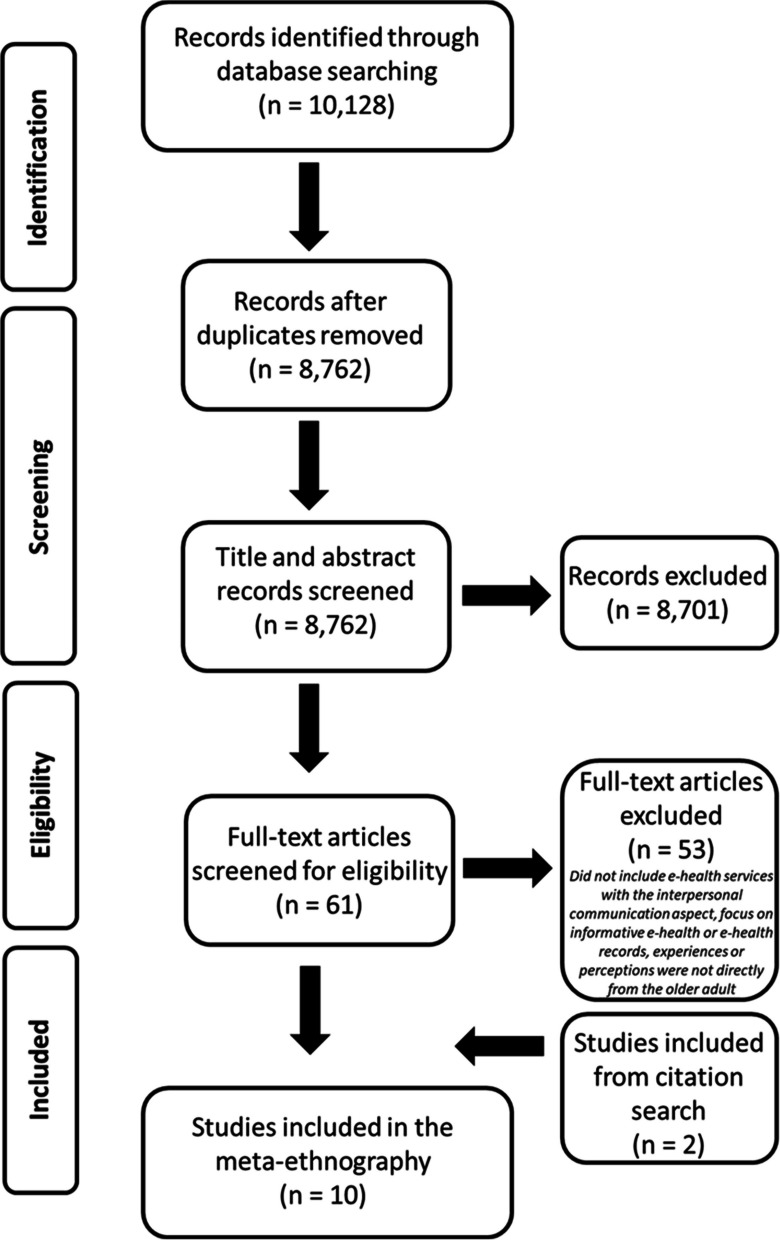
PRISMA flow chart of the search strategy

**Fig. 2:**
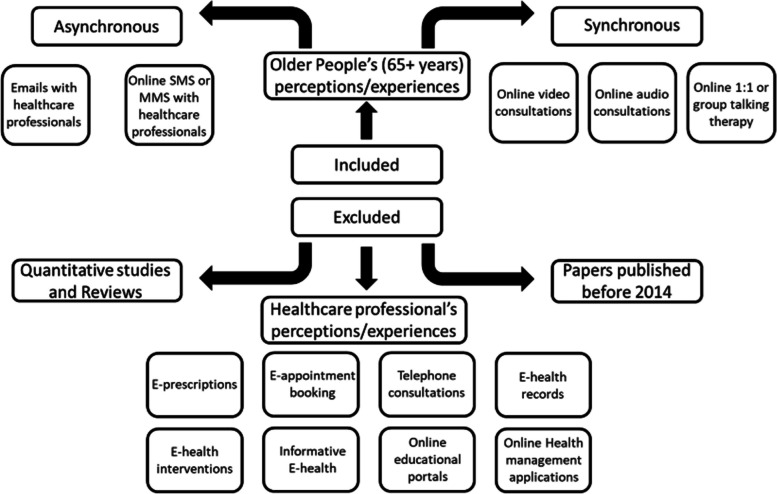
Inclusion and exclusion criteria flow chart

A systematic search was conducted on six databases: MEDLINE, CINAHL, PsycArticles, PsycInfo, ASSIA, and British Nursing Index. Search terms relating to ‘older adults’, ‘e-health’, ‘technology’, and ‘communication’ were utilised (see Table 1). Date limits were applied to all searches, with only studies published after January 2014 being eligible. As previously mentioned, this meta-ethnography builds upon Kapadia et al.’s [21] systematic review of studies using a range of qualitative and quantitative methodologies, therefore, the date limit was applied so that the literature they reviewed was not included in this synthesis. Initial searches yielded 10,128 articles (Fig. 1.) After deduplication and screening of titles and abstracts by two authors (AA and FM) 61 articles remained. These were subjected to full-text screening by two authors (AA and FM). Fifty-three articles were excluded at this point as they did not fulfil the inclusion criteria (see Fig. 2). We included only English-language published papers in the search. Additionally, citation searches and ‘similar articles’ for each included paper were searched which yielded two additional studies. A total of 10 articles were eligible and included in the synthesis (See Table 2).

**Table 1:** Example of search string

	Platform: EBSCO
	Database: Medline
	Limits: 01/01/2014—present; Human; English
	# Results: 4755
1	(MH "Frail Elderly")
2	TI ageing OR AB ageing
3	TI old* OR AB old*
4	TI elder* OR AB elder*
5	TI senior* OR AB senior*
6	TI geriatric* OR AB geriatric*
7	TI aging OR AB aging
8	OR—1/7
9	(MH "Telemedicine")
10	TI e-health OR AB e-health
11	TI ehealth OR AB ehealth
12	TI m-health OR AB m-health
13	TI mhealth OR AB mhealth
14	TI "digital health" OR AB "digital health"
15	TI "health technolog*" OR AB "health technolog*"
16	TI telemedicine OR AB telemedicine
17	OR—10/16
18	(MH "Pharmaceutical Services, Online")
19	(MH "Video-Assisted Techniques and Procedures")
20	(MH "Appointments and Schedules")
21	(MH "Shared Medical Appointments")
22	(MH "Health Services for the Aged")
23	OR—18/22
24	(MH "Digital Technology")
25	(MH "Online Systems")
26	(MH "Smartphone")
27	(MH "Mobile Applications")
28	(MH "Computers, Handheld")
29	(MH "Computer Systems")
30	OR—24/29
31	8 AND 17 AND 23 AND 30

**Table 2:** An overview of the 10 studies included in the meta-ethnography

Papers	Aim	Main Theme(s)
Jakobsson et al., 2019 [34]	To investigate experiences of using eHealth in contact with health care among older adults with cognitive impairments	1) Using well-known technology in a familiar way and environment 2) Interpersonal relationships with healthcare
Vergouw et al., 2020 [44]	To identify the needs, barriers, and facilitators amongst community-dwelling older adults (60þ) suffering from one or more chronic health conditions, in using online eHealth applications to support general practice services	1) Personal contact 2) Non-familiarity with online services 3) Difficulty using applications 4) Convenience and quick
Lindberg et al., 2021 [32]	To describe older people's perceptions of caring relations in the context of rural eHealth. To explore how such relations can facilitate engagement in digital primary healthcare services	1) Importance of in-person caring relations 2) Patient-nurse caring relations
Iyer et al., 2021 [41]	To examine the feasibility and acceptability of telemedicine visits for primary care and geriatrics consultation in complex older patients with a higher proportion of cognitive impairment	1) Set-up and useability 2) Satisfaction with visits
Johnson et al., 2021 [42]	To describe the key factors that could serve as barriers or facilitators to active surveillance of low-risk skin cancers using mHealth technology in older adults. To assess the attitudes and beliefs of participants, defined by the Theory of Planned Behaviour as a person's evaluation of a behaviour of interest and appraisal of whether important peers or society would accept that behaviour, respectively	1) Relationships between patient-provider 2) Social support 3) Knowledge of personal health issues
Loza et al., 2021 [45]	To explore the elderly's healthcare experiences during the lockdown and the problems that may have arisen regarding accessibility to the healthcare system and emerging adaptations to medical care	1) Access to regularly scheduled consults 2) Emergency consultations 3) the role of information and communication technologies
Pan et al., 2021 [13]	To understand older adults' perception of mHealth services and to discover the barriers that older adults face in the initial adoption of mHealth apps	1) Risk of privacy leakage 2) Interface of the application
Watt et al., 2022 [46]	To describe barriers and facilitators experienced by people accessing and providing virtual care in a geriatric medicine clinic	1) Impact of COVID-19 2) Complexity of virtual care 3) Uncertain accuracy 4) Inequity in access
Rochmawati et al., 2022 [47]	To explore the acceptance of health technology among older people who receive primary health care in Indonesia. To explore older people patients who use primary healthcare facilities for the acceptance of health technology as a means of home health monitoring	1) Demand of care 2) Resistance and openness 3) preference for home health monitoring
Mao et al., 2022 [43]	To investigate the top barriers to telemedicine visits from the perspectives of older adults with differing socioeconomic backgrounds and primary spoken languages in two independent living facilities in Northern California	1) Limitations of video visits 2) The process of learning 3) Desire for in-person/on-demand support

Papers were excluded if the participants were aged below 65 years, did not include older adults' perceptions or experiences (e.g., family, carer, or healthcare professional’s perceptions or experiences), did not include a qualitative element, it was published before 2014 or did not include an e-health service which had an interpersonal communication element. Many papers were identified which included older adults’ perceptions and experiences of e-health services, however, if there was not an interpersonal communicative element, these were excluded. Additionally, only primary qualitative research papers were included, therefore, all reviews were excluded, along with any quantitative research or reviews. Figure 2 illustrates different communicative e-health services that were deemed to have an interpersonal communicative element, whether it be asynchronous or synchronous.

### Quality appraisal

The Joanna Briggs Institute (JBI) critical appraisal tool for qualitative research was used to assess the quality of eligible studies [33]. It was selected as it is a standardised tool for assessing the methodological quality of qualitative studies including biases in the study’s design, conduct, and analysis. Two authors independently critically appraised each study; AA critically appraised all studies, and the second appraisal of each paper was divided between FM, HvM, and JA. All studies were included in the synthesis, regardless of their critical appraisal outcome, as their findings provided an important perspective to our research. Overall, most studies were clear in their philosophical and methodological stance, however, many did not address the researcher’s influence on the research, nor provided a statement that located their cultural or theoretical views, with only one study outlining their stance [34]. Only one study explicitly located the researchers culturally or theoretically [32]. Thus, all the studies proved to be sound in their methodological design, but few provided a reflexive account. A lack of reflexive practice (or not mentioning that in papers) within qualitative research is a common occurrence [35], despite the importance transparency brings to the research process [36].

### Data synthesis

This meta-ethnography followed stages 1–6 of the eMERGe guidelines [37]. Stage 1 explores if there are any other meta-ethnographies on the chosen topic and if a meta-ethnography is needed by identifying the gap in the evidence or providing a justification as to why it may need updating. Stage 2 is when the reviewers decide what is relevant; the search strategy is informed by the aim of the research and choosing what search approach to adopt, including any search limits to apply. For this meta-ethnography, a comprehensive search was chosen so all available studies were screened.

Stage 3 consists of close and repeated reading of the eligible articles and extracting relevant data (aims, methodology, and sample characteristics [38]) using a bespoke Excel data extraction form. AA led both the reading of and recording of interpretative metaphors, including first- and second-order constructs from the studies. First-order constructs refer to primary data from the studies such as direct quotations, whereas second-order constructs refer to the authors' interpretations [39], for example, metaphorical themes and concepts. Additionally, France et al. [37] defined third-order constructs as the reviewer’s higher-order interpretations which are developed from a tertiary analysis of the first- and second-order constructs.^1^ First- and second-order constructs comprised quotes extracted from the papers -either direct participant quotations or the authors' interpretations of their findings—which were copied into the data extraction form as separate constructs (see Additional file 1).

Stage 4 focuses on how studies are related and identifies relationships between key themes and findings from across the studies. This helps to develop overarching concepts. All the studies were related in terms of exploring older adults’ experiences and/or opinions on communicative e-health services. Most studies referred to exploring the barriers and facilitators to e-health services, with many of the e-health services being virtual visits/video appointments.

Stage 5 translates the studies into one another by following Sattar et al.’s [40] guidance; each theme from the eligible studies was compared to the others to check for commonalities. We found many overlapping themes among the papers. These commonalities were developed further into conceptual categories, due to clear differences and similarities between the themes, thus, producing third-order constructs. Studies were arranged in chronological order, starting with the oldest study first. The rationale for this decision was to follow the timeline of advances in e-health services which may have influenced individuals’ perceptions and experiences. Five studies included participants who had never used communicative e-health services. Differences in individual study findings were explained by the context of the studies, such as country of origin and older adults’ health conditions. Reciprocal translations of concepts were the most frequent type of translation.

Stage 6 involved synthesising the translations of the studies to provide deeper inferences across the findings. Despite reciprocal translations being frequent across the studies, the first- and second-order constructs provided a more in-depth understanding of older adults’ perceptions and experiences. Thus, a line of argument synthesis was conducted, as the authors deemed it more appropriate, whereby new and in-depth explanations as to why older adults may or may not engage with communicative e-health services were derived. All studies were used within the synthesis, despite a few only covering a small number of the overall concepts (see Table 3 for the concept matrix). These studies added conceptual depth as they covered topics that were highly reported throughout all studies. Third-order constructs were developed by translating first- and second-order constructs directly from studies. The synthesis emphasises the direct and indirect links between the identified themes and provides a new interpretation and explanation of older adults’ experiences and perceptions of communicative e-health services.

**Table 3:** Concept Matrix Across Eligible Studies

	**Jakobsson et al., 2019 **[34]	**Vergouw et al., 2020 **[44]	**Lindberg et al., 2021 **[32]	**Iyer et al., 2021 **[41]	**Johnson et al., 2021 **[42]	**Loza et al., 2021 **[45]	**Pan et al., 2021 **[13]	**Watt et al., 2022 **[46]	**Rochmawati et al., 2022 **[47]	**Mao et al., 2022 **[43]
Health barriers	x			x	x					x
Support networks	x		x		x	x		x	x	x
Application interface/design	x	x			x		x			x
Digital literacy		x			x	x		x	x	x
Online security		x	x		x		x			
Access to technology and the internet				x			x	x	x	
Lack of awareness	x	x					x	x		x
Relationship with healthcare provider(s)	x	x	x		x	x		x		
In-person preference	x	x	x	x	x	x	x	x	x	x
Convenience		x	x	x	x	x			x	

Two additional files show the initial analytic process (see Additional file 1) and tables providing overviews of first- and second-order constructs from the eligible studies (see Additional file 2).

### Reflexivity

The first author, AA, plays an active role in supporting older adults to use digital technology and has personal experience with assisting older family members with their devices, along with other older adults. From this experience, they have some presumptions about how a lot of older adults have difficulties with technology and lack the necessary digital skills and language to use it. However, this has fed into but is also separate from the current review. Therefore, whilst authors may have their own preconceived biases about the topic area and their personal experiences with the services, these may have impacted the overall research: HvM is 63 years of age (i.e., an older adult himself). AA’s epistemological stance is interpretivism as they recognise that humans are social beings and they interact with the real world, thus everyone will have their own subjective experience whereby, we as researchers, must try to interpret their understanding and consolidate the information. While HvM and JA’s epistemological perspective is critical realist.

## Results

All the studies were related in terms of exploring and providing older adults experiences and/or perceptions of communicative e-health services. Most studies explored barriers and facilitators to e-health services, with many e-health services being virtual visits/video appointments, whilst two studies mentioned the use of WhatsApp. Methodological approaches were compared across the studies, with 7 of 10 studies using semi-structured interviews. Additionally, as many of the studies were published after 2020, it was unsurprising that COVID-19 was a reoccurring topic.

The 10 eligible studies were conducted in seven countries: Sweden [32, 34]; USA [41–43]; UK [13]; The Netherlands [44]; Argentina [45]; Canada [46]; and Indonesia [47]. The sample size ranged from nine to 39 participants, however, three studies [43, 45, 46] also recruited healthcare providers and/or caregivers as participants. The gender distribution across the studies was balanced, with a55% of the aggregate number of participants across the studies being female (Range 86.3%- 8.7%). Most studies focused on virtual and/or video consultations. Other forms of communicative e-health services included virtual health rooms, remote patient monitoring systems, WhatsApp, direct messaging, and teleconsultations; these services included synchronous and asynchronous communication. Semi-structured interviews were the most favoured method of data collection [32, 34, 42–47]. Regarding analytical methods, thematic analysis [13, 42, 43, 47] and qualitative content analysis [32, 41] were the most frequent. Furthermore, three studies utilised a mixed methods approach [13, 41, 43]. Table 4 provides an overview of the demographics and characteristics of the selected studies.

**Table 4:** Eligible studies demographic

Study	Country	Participants	Gender composition (% male)	Type of communicative e-health service	Experience in using communicative e-health services?	Methodology	Analysis
Jakobsson et al., 2019 [34]	Sweden	9	66%	one-way-use / interactive e-health	No	Semi-structured interviews	Grounded theory
Vergouw et al., 2020 [44]	The Netherlands	19	53%	e-health application to support GP services, e-consultations, e-appointments	Yes	Semi-structured interviews and	Verbal analysis
Lindberg et al., 2021 [32]	Sweden	19	36.84%	e-health applications – remote patient monitoring system	Yes	Semi-structured interviews	Qualitative content
Iyer et al., 2021 [41]	USA	43	90.70%	video consultation	Some	Case study and survey^b^	Qualitative content
Johnson et al., 2021 [42]	USA	33^a^	46%	Skin disease active surveillance / mHealth	Yes	Semi-structured interviews	Thematic analysis
Loza et al., 2021 [45]	Argentina	39	18%	video calls / WhatsApp / teleconsultations	Some	Semistructured interviews	Iterative processing
Pan et al., 2021 [13]	UK	30	57%	mHealth applications, online consultations, video calls, and	Yes	Questionnaires and interviews	Thematic analysis
Watt et al., 2022 [46]	Canada	20^a^	35%	videoconference-based assessments	Some	Semi-structured interviews	Framework analysis
Rochmawati et al., 2022 [47]	Indonesia	11	36.70%	WhatsApp, Home health monitoring	Some	Semi-structured interviews	Thematic analysis
Mao et al., 2022 [43]	USA	15^a^	13.30%	virtual visits	Yes	Survey and semi-structured	Thematic analysis

^a^Sample was made of older adults and other populations e.g., caregivers, healthcare providers

^b^Mixed-method study, both qualitative and quantitative data collected

In total, 10 reoccurring concepts were developed (see Table 4). All concepts were present in three or more studies. The results section describes the final line of argument synthesis which comprises the authors’ interpretations of their findings (third-order concepts). An overview of the constructs covered in each study is provided in Table 3.

Please refer to Additional File 3 for a summary table including additional information.

**Fig. 3:**
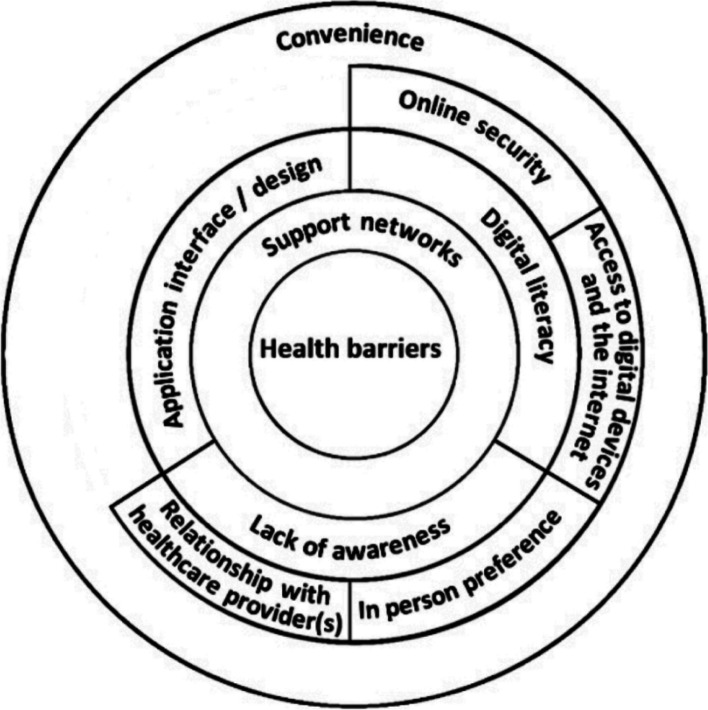
A model for conceptualising the barriers and facilitators of older adults adopting communicative e-health services and the interlinking connections between the factors

The model represents a ripple effect of the concepts, with the centre rings having an impact outward on the concepts, as well as from the outer rings inwards. Health barriers were identified as causing issues with adopting the device and utilising communicative e-health services, such as poor motor skills, and visual and hearing difficulties. However, despite this, support networks were useful in helping with accessing the devices, as well as communicative e-health services. Support networks can assist in increasing older adults’ digital literacy skills which, in turn, improves online security awareness as well as access to other digital devices and the internet. Additionally, support networks help to increase older adults’ awareness of what communicative e-health services are available. However, even after being aware of communicative e-health services available, they have been met with pessimistic views. Overall, most older adults appeared to have negative experiences and perceptions of online e-health services as they reported not being able to build a relationship with their healthcare providers virtually and so they preferred to see clinicians in person. There were, however, some exceptions to this. Finally, support networks increased older adults' understanding and navigation of communicative e-health services. Lastly, having support from their social network helped older adults use services at their convenience and so see the benefits of communicating with health services in this way Fig. 3.

The concepts derived from the findings of the studies are: 1) health barriers, 2) support networks, 3) application interface/design, 4) digital literacy, 5) lack of awareness, 6) online security, 7) access to digital devices and the internet, 8) relationship with healthcare provider(s), 9) in-person preference and 10) convenience.

### Health barriers

Physical and mental health impairments appear to be a barrier for older adults using digital technology [34, 42], more so when it comes to accessing communicative e-health services as the studies illustrate [41, 46]. Older adults are more likely to experience cognitive impairments, hearing, and visual difficulties as well as limited motor skills, making it difficult to engage with communicative e-health services [34, 42]. Moreover, hearing difficulties were a common factor in older adults’ negative experiences, especially those using virtual services [41, 46]. Whilst others found that their hearing impairment was compensated for in video visits with their clinician as they could increase the volume, something they are unable to do during an in-person visit [41].

### Support networks

Support networks play a key role in older adults accessing communicative e-health services. Many depend on family members to assist with using digital technology due to older adults’ unfamiliarity with the devices [41–43]. Older adults perceived that younger family members were ‘tech savvy’ and had a better understanding of how to use and set up communicative e-health services [45]. The set-up process for communicative e-health services poses difficulties for older adults [32, 43]. More so for older adults at home whose primary language is not English; language barriers impact their ability to set up their account and telemedicine video, with the added difficulty of needing a family member, caregiver, or healthcare professional to be available to act as their translator during appointments [43].

Support networks appeared to play an influential role in the initial uptake of applications for older adults; families and caregivers played an important role in encouraging older adults' initial adoption [47]. In addition, older adults showed a preference for clinicians to help set up communicative e-health services during a clinic visit [32] whilst also expressing concerns regarding maintaining contact with health services without having support [34, 42] and feelings of resistance in asking for support [34]. This perceived dependence on families could be a barrier to older adults using communicative e-health services. For example, some appeared to rely on family members to send them healthcare information rather than searching for it themselves [47].

### Application interface and design

Older adults reported that the communicative e-health services’ interface can be complicated [44], thus requiring them to seek out additional help. This suggests that the design of the application impacts perceived ease of use [13]. Therefore, for older adults to perceive a communicative e-health service as easy to use and for them to want to use it, it must be simple and straightforward [42, 44]. As such, easy-to-read instruction manuals should be available for older adults and other individuals with limited digital skills and experience [34]. The instructions should cover basic information such as set-up, page structure, and how to connect with your healthcare provider. Alternatively, older adults may prefer more familiar platforms to communicate with their healthcare providers rather than specific e-health services [43, 45]. It has been suggested that using familiar platforms may increase older adults’ usage of communicative e-health services and thereby increase their access to healthcare [43].

### Digital literacy

Many older adults stated their digital literacy levels were low, which hindered their ability to use communicative e-health services [41, 44, 46, 47]. There are two aspects to digital literacy 1) older adults’ ability to use communicative e-health services and 2) their capacity to understand how to use communicative e-health services.

Ability to use communicative e-health services: Whilst some older adults reported limited health technology skills, they were able to use basic applications, such as WhatsApp [47], or make a phone call (54). Older adults reported preferring to phone the doctor and arrange a face-to-face appointment [46]. Their limited digital skills could be a consequence of not growing up with advanced technology such as smartphones and tablets [44]. Some older adults expressed concerns about being too old, it is too late to learn digital skills [46], and that it was too difficult for them to understand how to use the services [44]. Nevertheless, many older adults reported wanting to improve their digital skills as they understood they needed these skills to use online health services [44, 46].

*Capacity to understand:* Using the e-health services was reported by older adults as being frustrating when things did not work as expected due to their low levels of digital literacy [44]. Additionally, one older adult spoke about how they had bought a smartphone but not was not accompanied by a user manual [43]. Moreover, some reported being fearful of making mistakes whilst using communicative e-health services [44], such as sending messages to the wrong healthcare professional, and people outside their immediate care being able to access and read their health records. Regarding the devices they use, some older adults preferred computers and laptops to tablets due to the screen size [44]; corroborating the previous point of poorer manual dexterity or other impairments impacting the physical use of devices [42]. Those with poor eyesight need an application that is easy to navigate and that they can see without difficulty [43]. Some reported issues in uploading and sending photographs of their problems to their healthcare providers [42].

### Online security

With limited digital literacy, online security is perceived as a risk for older adults; this is reflected in their perceptions of online security across different devices, with one individual considering their laptop as more secure than their mobile phone [42]. Additionally, older adults expressed concerns about their online privacy and the lack of trust they have in communicative e-health services, [13, 42, 44]. Consequently, the risk of privacy leakage decreased their perception of communicative e-health services as useful [13].

### Access

Findings suggest some older adults have issues when accessing devices and the internet [46] meaning they are unable to benefit from communicative e-health services and are limited to using traditional methods. On the other hand, some older adults may have a device but poor internet speed [13] which can make using communicative e-health services frustratingly slow to use and may discourage them from using e-health services. However, some findings suggested that even when older adults were able to access technology for communicative e-health services, this did not equate to the successful use of applications [34]. In addition, some older adults experienced not having enough storage space on their devices to download the communicative e-health application [13] and reported uncertainty when deleting applications on their phones to make room for new communicative e-health applications [13].

### Lack of awareness

Older adults were often not aware of e-health services and what they had to offer [34]. The need to be informed about e-health services availability was expressed by older adults with importance placed on how they are informed; traditional methods such as letters, information sheets, and poster advertisements were preferred [34]. In contrast, when older adults were aware of the communicative e-health services available to them, they reported being unfamiliar with using digital technology [43, 44]. This links back to digital literacy and being unable to navigate applications, and so needing additional support to use them. Nevertheless, older adults wanted better access to digital technology and opportunities to improve their awareness and skills [46].

### Relationship with healthcare provider(s)

The relationship with healthcare professionals is a facilitator to older adults adopting communicative e-health services [32, 34]. Older adults shared how trusted nurses played an essential role in helping them to use digital applications [32]. Therefore, having a strong established patient-healthcare provider relationship could encourage older adults to try out new communication methods, as they appreciate the time taken by healthcare providers. However, it was noted that the relationships with clinicians could also be a barrier to older adults using communicative e-health services [42], if the relationship was negative or weak relationships, resulting in a lower sense of trust and more unwillingness to engage with the technology. Pre-existing relationships were seen as vital to older adults having a positive experience with communicative e-health services [46] as they trusted the healthcare provider and believed the clinician had a better understanding of their health issues.

### Preferences to in-person contact

The biggest barrier to older adults adopting communicative e-health services was their preference for in-person contact (face-to-face) which was reported in all the reviewed studies. Older adults reported that being able to communicate with their doctors in person provides a personal and human connection between patient and physician, offers the chance to ask questions right away [44], and allows patients to see their expressions [13, 43]. Older adults reported that they did not have an issue with arranging their GP visits via telephone or online, however, many preferred their appointment to be in-person [43].

Having in-person appointments made older adults feel more comfortable [13]. Some older adults expressed how they were worried that during a virtual appointment, clinicians would not conduct routine checks such as measuring blood pressure [46]. They preferred the doctor to be able to see and touch them if they had any health issues so they could be examined sufficiently rather than solely through verbal descriptions and video calls [43]. Additionally, older adults believed that virtual appointments were not as comprehensive as seeing a doctor in person [43, 45, 46]; they did not understand how it was possible to assess someone virtually, however, this could be linked to their limited digital skills and understanding of technology.

This preference for face-to-face contact may link to other barriers and facilitators previously identified such as digital literacy, application interface, support networks, and convenience. A few older adults preferred in-person communication with their healthcare provider as they lived close to the clinic and did not see the need to do this virtually [44, 47]. Those who have physical and mental impairments, as well as poor digital skills, may prefer to see a healthcare professional in person as they believe they will receive a more comprehensive check-up [43, 45, 46], as well as not having to go to the trouble of asking for support in setting up their communicative e-health application.

### Convenience

The convenience of using communicative e-health services to connect with healthcare professionals is a positive factor for older adults [41, 44, 45, 47], as was recognising how it may benefit healthcare workers [32]. Older adults shared their thoughts on how communicative e-health services could mean they could have more frequent connections with clinicians and how this could be beneficial in both strengthening their relationships and having regular check-ups [45]. It proved to be time efficient and in turn, cost-effective for many older adults who have far travel to their doctor’s clinic [41, 42, 45]. Older adults could connect with their clinicians online from their homes, attend from any location, and have appointments arranged on dates and times to fit into their schedule [44]. Additionally, older adults tend to be prescribed more medication than younger people, therefore, communicative e-health services allow them to communicate virtually with their doctor which eases the difficulty of attending the clinic or phoning up for a prescription [45].

The convenience, however, of being able to connect with healthcare professionals is only noted by older adults who have *previously used* these communicative e-health services [32, 41, 42, 44, 45, 47].

### Line of argument synthesis

The line of argument synthesis provided a pathway to understanding factors that act as barriers and facilitators to older adults, using communicative e-health services and how these interlink with each other. Figure 3 illustrates how each theme may link to the other. In the centre are health barriers; the findings suggest that making the technology more accessible may prevent the outer ring factors from becoming a barrier for older people. The convenience of communicative e-health services encompasses all concepts, providing that they are perceived positively. For example, having support networks to assist with health barriers that hinder the use of communicative e-health services, will in turn support them to use an application with a difficult interface, help to improve their digital literacy, and make them aware of other services available to them. The findings also highlight that having an established relationship with their healthcare providers and a preference for in-person contact appeared to be important facilitators and barriers, respectively.

## Discussion

The synthesis highlighted several barriers and facilitators to older people adopting and using communicative e-health services. Access to a support network appears to be vital for the adoption process, due to the knock-on effect it has on other barriers by reducing the impact of other factors being a barrier for older adults. Health barriers were reported by some older adults as an issue with both using the digital device itself as well as the communicative e-health services [34, 41–43, 46]. Moreover, older adults who participated in the reviewed studies (many of whom had more complex needs) discussed their limited digital literacy as being a barrier to using these services, along with a lack of knowledge of online security [13, 32, 42, 44] and complicated interfaces reinforcing the limited usage of communicative e-health services [13, 34, 42, 44]. Furthermore, limited access to, or having out-of-date digital devices and internet providers proved to hinder their ability to utilise communicative e-health services [13, 41, 46, 47]. Older adults' lack of awareness of what services are available to them adds to their limited usage. Interestingly, interpersonal relationships between older adults and clinicians were viewed as a facilitator to adopting communicative e-health services as a level of trust had already built up before moving to online communication [32, 34, 42, 44–46]. Additionally, convenience proved to be a facilitator to engaging with communicative e-health services as it appeared to limit time and effort in travelling to clinics as well as being able to have access anywhere and anytime [32, 41, 42, 44, 45, 47]. Despite this, most older adults expressed a strong preference for seeing their healthcare providers in person rather than using communicative e-health services, as reported in all the studies.

Regarding support networks, older adults who participated in the studies discussed the different roles people played in their adoption process [48]. Younger family members were perceived to have a better understanding of how to set up and use communicative e-health services. Older adults’ children provided advice and assistance on learning how to use the services and devices, and their spouses were there to support them, whilst their grandchildren influenced their overall adoption.

New factors, which have not been reported before, appear to specifically have an impact on communicative e-health service adoption and usage amongst older adults, with the most important being the need for an interpersonal relationship between patient and healthcare provider(s). Opposing factors were shown to impact the adoption of communicative e-health services, with older adults having a strong preference for in-person communication, whilst others expressed how convenient these services are. Moreover, the synthesis identified how the factors interact with each other which could further decrease or increase the likelihood of using communicative e-health services. The factors identified in the current review support findings from previous literature within the area of older adults and e-health services, such as chronic illness [17–19], the interface and design [11–13], poor digital skills [9, 16, 19–21], and limited understanding of or concerns regarding privacy and security online [8, 13, 15, 16]. Additionally, facilitating factors highlighted by our review corroborate previous literature findings, such as having support networks [9, 22–25], recommendations by healthcare providers [26], and having access to technology and the internet [10, 27].

Implications for practice include making sure healthcare providers can support older adults to engage in their communicative e-health services if they make sure that it is simple to use or to use familiar software. Moreover, healthcare practices could assign someone with experience in explaining technology to older adults or provide training to make their online services more accessible. If using a new application, a set of easy-to-read instructions should be available, so the older adult can navigate the platform without complications and with minimal support, whilst providing the option for older adults to access in-person support from healthcare providers if needed. Whilst providing a simple application, along with adequate support, healthcare providers must actively advertise and promote services that may be of interest to older adults as they may not be aware of what is available. Additionally, healthcare providers should prioritise appointments that need in-person examinations, such as having measurements or physical assessments, over encounters that are more suited for communication only which could be done online. The findings suggest that a hybrid model of primary healthcare may be the best option. A hybrid model, with a mixture of face-to-face and online communication, will enable older adults to build that initial relationship with healthcare professionals when meeting in person, whilst benefitting from the positive aspects of communicative e-health services.

The current synthesis is built upon Kapadia et al.’s [21] 2015 systematic review of issues that impact the adoption of information and communication technologies (ICT) in the aged care sector. We narrowed the focus to older adults’ perspectives and experiences with communicative e-health services. There were some similarities between the factors found in both reviews which may influence the adoption of ICT within aged care and communicative e-health services amongst older adults. Similarities included health barriers, privacy and security, family, friends, and healthcare provider influence, preference for person-led care, and technology issues such as the ICT’s design and functionality. Kapadia et al.’s findings highlight the importance these factors play within the adoption process of general e-health services, whilst the current synthesis has identified further factors that appear to have an influence specifically on communicative e-health services, such as older adults needing to have a strong interpersonal relationship with their healthcare providers before agreeing to communicate with them online. Additionally, there are factors within Kapadia et al.’s review that are of equal significance concerning communicative e-health services, such as cost and reliability. These differences may be explained by the accessibility of technology and services and the range of affordability options now, compared to a decade ago. Finally, our synthesis adds to the overall topic area with direct perceptions and experiences from older adults themselves, rather than care providers, families, and organisations which may skew the general findings with their personal biases.

Comparatively to similar reviews within the area, a key difference that our synthesis highlights is the interactions between the factors, whereas other reviews report their findings as independent factors that are a barrier or a facilitator to adopting e-health services. Additionally, most reviews conducted were quantitative, evaluating e-health interventions, which ignores the growing body of qualitative literature about older adults’ use of e-health services. Our synthesis of these qualitative studies revealed that older adults felt it was important it is to have an interpersonal relationship with their healthcare provider before communicating with them via e-health services, which otherwise would not have been able to be interpreted through quantitative results. Whilst many factors reported in our synthesis have been identified in the broader e-health literature, our amalgamation of older adults’ direct perceptions and experiences has highlighted the importance of how factors interlink with each other, which has not been identified previously.

### Limitations

Some older adults in the studies reviewed had no experience with communicative e-health services. Future reviews may consider looking at the level of experience of communicative e-health services independently to identify more specific barriers and facilitators of usage and adoption, such as older adults with no, some, or lots of experience. Likewise, a comparative exploration of the perceptions of communicative e-health services from older adults who do and do not use them could provide further insight into the determinants of using these services.

Finally, a lack of reflexive practice in the eligible studies makes it unclear whether the researcher's theoretical stance and beliefs influenced the outcome of their research, thus, like much research, this work may have been subjected to potential researcher bias. Clearly outlining any overlapping relationships and beliefs between the researchers and participants increases the credibility of the study whilst also gaining a better understanding of the results [49].

## Conclusions

The review is the first to synthesise vulnerable older adults’ experiences and perceptions of e-health services and specifically focus on ways to improve communication between patients and healthcare providers. Overall, the review adds to our understanding of why older adults are reluctant to use communicative e-health services, based on their experiences and perceptions, with many reporting barriers to engaging with online services, which may increase avoidance tendencies. The determinants of older adults adopting and using communicative e-health services are complex, with many interrelated factors, the overarching one being access to support. Many negative experiences are related to a lack of support networks and poor digital literacy, along with complicated application interfaces. For some older adults, their physical and mental health require them to have assistive technology and/or support to use the technology device. Whereas others with poor digital literacy and a lack of awareness of the communicative e-health services available need the initial support to provide the opportunity to use these independently. Having access to or providing the correct support for older adults, the barriers faced can be minimised, and their access to services may, in turn, increase.

### Supplementary Information

**Additional file 1. **Data extraction & preliminary synthesis.

**Additional file 2. **A list of first and second order constructs.

**Additional file 3: Table 5. **Summary table with additional information.

## Data Availability

The datasets used and/or analysed during the current study are available from the corresponding author upon reasonable request.

## Footnotes

1 Please refer to France et al. [37] or Sattar et al.’s [40] paper for further details and explanation on meta-ethnography terminology.

## Glossary

Digital divide: The difference in access to technology
Digital inequalities: The differences in access to technology and the inequalities within individuals who have access
Digital literacy: The ability to find, evaluate, and communicate information using information and communication technologies
E-health: The use of information and communication technologies to promote access to quality healthcare services
Telemedicine: The remote diagnosis and treatment of patients via telecommunications technology
