# Supplementary material for: What are the determinants of older people adopting communicative e-health services: a meta-ethnography
Source: BMC Health Serv Res. 2024 Jan 11;24:60. doi: 10.1186/s12913-023-10372-3 (PMC10785477; doi:10.1186/s12913-023-10372-3)
Supplement: Supplementary file 1 — Additional file 1. Data extraction & preliminary synthesis. [file 12913_2023_10372_MOESM1_ESM.pdf]

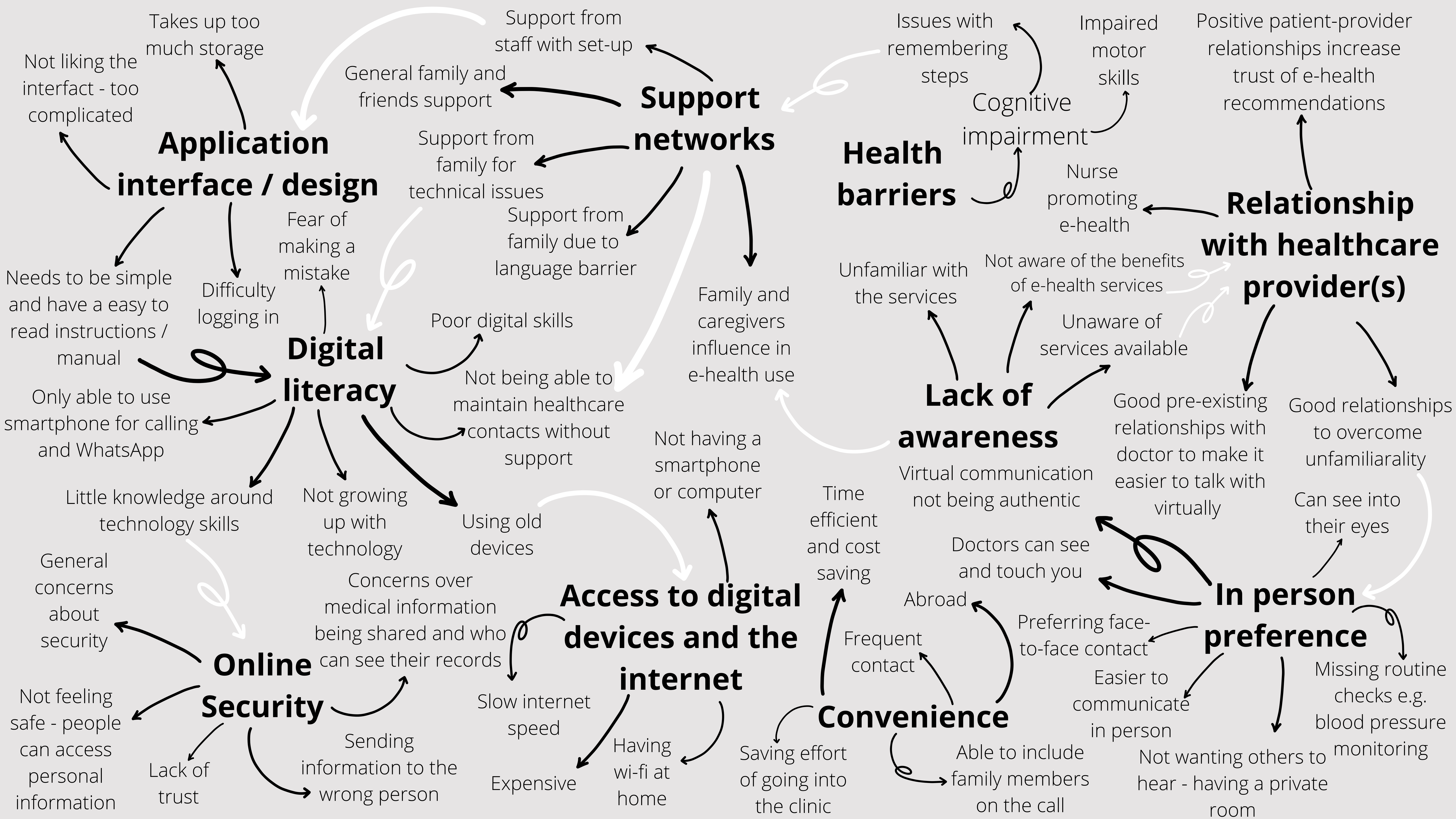
Application interface / design
Takes up too much storage
Not liking the interface - too complicated
Needs to be simple and have a easy to read instructions / manual
Difficulty logging in
Fear of making a mistake
Support networks
Support from staff with set-up
General family and friends support
Support from family for technical issues
Support from family due to language barrier
Family and caregivers influence in e-health use
Digital literacy
Poor digital skills
Not being able to maintain healthcare contacts without support
Only able to use smartphone for calling and WhatsApp
Little knowledge around technology skills
Not growing up with technology
Using old devices
Health barriers
Cognitive impairment
Issues with remembering steps
Impaired motor skills
Relationship with healthcare provider(s)
Positive patient-provider relationships increase trust of e-health recommendations
Nurse promoting e-health
Good pre-existing relationships with doctor to make it easier to talk with virtually
Good relationships to overcome unfamiliarality
Lack of awareness
Unfamiliar with the services
Not aware of the benefits of e-health services
Unaware of services available
Virtual communication not being authentic
In person preference
Can see into their eyes
Doctors can see and touch you
Preferring face-to-face contact
Easier to communicate in person
Not wanting others to hear - having a private room
Missing routine checks e.g. blood pressure monitoring
Convenience
Time efficient and cost saving
Abroad
Frequent contact
Saving effort of going into the clinic
Able to include family members on the call
Access to digital devices and the internet
Not having a smartphone or computer
Slow internet speed
Expensive
Having wi-fi at home
Online Security
General concerns about security
Concerns over medical information being shared and who can see their records
Not feeling safe - people can access personal information
Lack of trust
Sending information to the wrong person

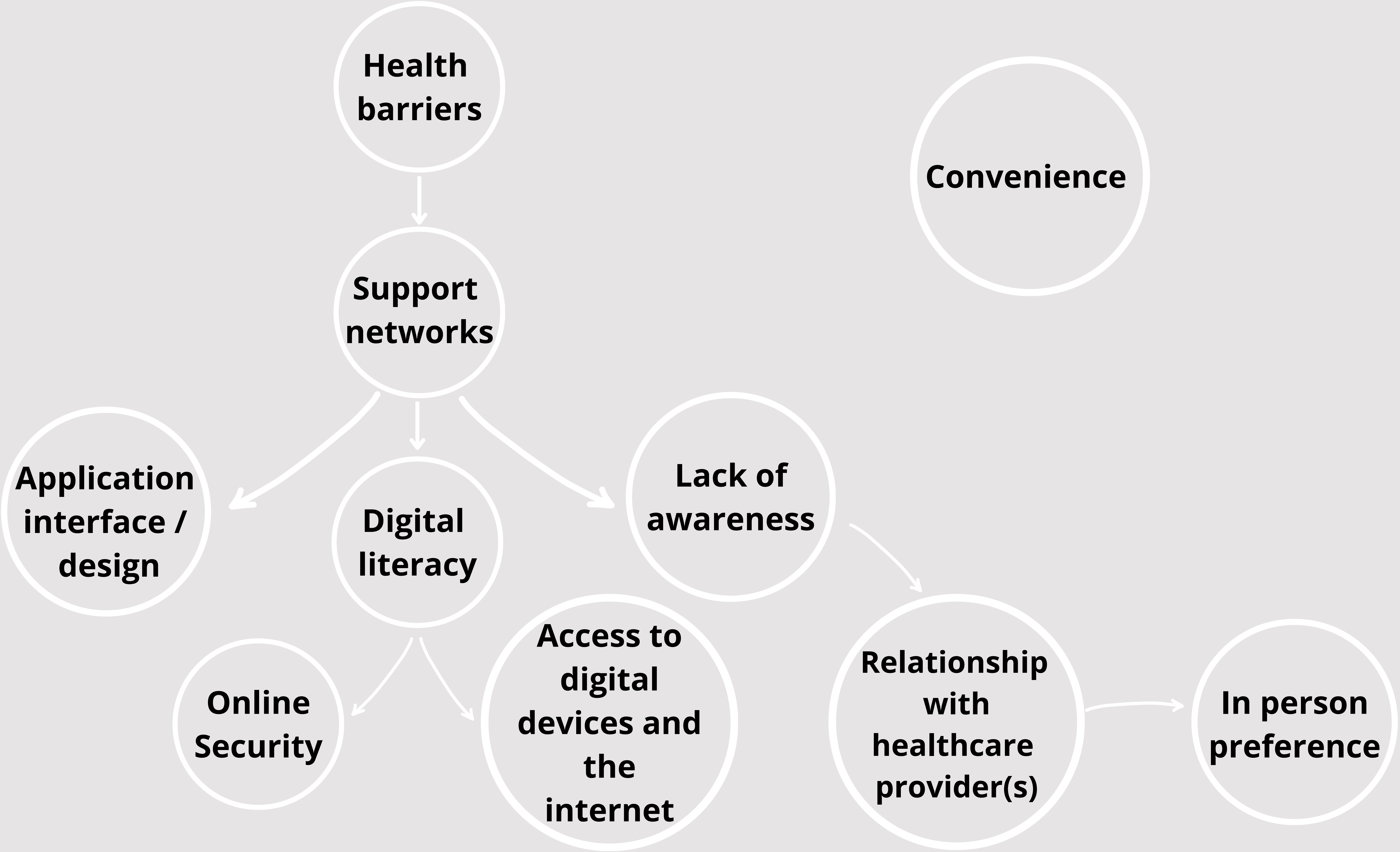
Health barriers
Support networks
Convenience
Application interface / design
Digital literacy
Lack of awareness
Online Security
Access to digital devices and the internet
Relationship with healthcare provider(s)
In person preference

Health barriers

challenging for older adults to access virtual care, including hearing impairment, cognitive impairment and need for translation services - Watt et al., 2022

Complexities in caring for older adults such as the presence of multiple chronic conditions (e.g. hearing impairment and cognitive impairment), polypharmacy, frequent need to obtain collateral history, and heterogeneity in patient and caregiver digital literacy, among others, make virtual care challenging - Wats et al., 2022

I don't have the dexterity of... people... I don't like...texting - Johnson et al., 2021

Challenges with hearing impairments but was helped during video visits - Iyer et al., 2021

Poor use of hands - Mao et al., 2022

Impaired motor skills and difficulty with the touch functioning - Jakobsson et al., 2019

Support networks

"As long as my kids and my wife are alive, it would be okay... Because I'm not a techy guy...So I'd be okay with it if I've got somebody to help me" Johnson et al., 2021

"If it seemed too complicated to do, I mean there are enough people around... There are two people in the family that are in IT and are extremely savvy and could help and can teach and can support me" Johnson et al., 2021

"I was here [at Healthcare Centre 1] and one of the staff showed me and connected it so that they could synchronise it with my phone. You had to download some 'app'. (...) I cannot do that. She [a local Registered Nurse] did that" Lindberg et al., 2021

important to consider the presence or absence of support systems such as caregivers - Johnson et al., 2021

some of the participants stated that they felt resistant to asking for support - Jakobsson et al., 2019

"I do not browse by myself, usually my children will do the search if I experience symptoms or health complaints then they will send the information to me" Rochmawati et al., 2022

Having a caregiver present to facilitate virtual care was identified as a key facilitator of videoconference-based virtual care for patients - Watt et al., 2022

"I need a person to sit down with me next to my computer to help me set up my account: here's the icon you click on, the name of your account, where you keep your password, how you enter and use it...I need personal help" Mao et al., 2022

Some participants expressed worries about not being able to maintain their health care contacts without the support - Jakobsson et al., 2019

They also highlight family support, as younger family members have better use of these technologies - Loza et al., 2021

"If I use English, it will be very hard. I am comfortable with computers, and am willing to give it a try...I hope there is someone who speaks Chinese to help with technology" Mao et al., 2022

Openness to technology can be persuaded by caregivers. The availability of support for older people is important in health technology adoption among older people - Rochmawati et al., 2022

for the participants who did not speak English as their primary language, setting up telemedicine visits involved an added layer of difficulty as most of the instructions are in English - Mao et al., 2022

Application interface / design

"Accessibility is important to me. You should be easily able to log on using your username and password" Vergouw et al., 2020

"I just don’t enjoy setting it up" Mao et al., 2022

important to consider the usability of the app - making it simple - Johnson et al., 2021

"Yes, how to operate the applications was all clearly indicated, there was nothing wrong there" Vergouw et al., 2020

Some stated that the structure of the page was unclear - Vergouw et al., 2020

difficulty with the log in and too many screens making things complex - Vergouw et al., 2020

"[The telemedicine platform is] very complicated -- much more so than Zoom. I have very poor vision and I’m old and it’s no good for me... I think just having help at the time I have to get on is the best or you should switch to a simpler system" Mao et al., 2022

Having sufficient instructions - Jakobsson et al., 2019

All participants stated that the applications have to be designed clearly and simply - Vergouw et al., 2020

"There are many steps to book the [telemedicine] app, I have received a lot of information (eg. email) on how to connect. I feel like I am not smart enough to persist through the whole [set of instructions]" Mao et al., 2022

the design of app interface will directly affect the perceived ease of use - Pan et al., 2021

Some noted that they are familiar with platforms they already use such as Zoom or WeChat and would prefer if their care providers switched to simpler platforms for telemedicine video visits - Mao et al., 2022

Digital literacy

"Yaa... the thing there is a problem related to digital knowledge. That is all, so if I have to open it or press certain keyboard to open something I cannot do it, I do not understand and obviously have very little knowledge" Rochmawati et al., 2022

"But we did not grow up with the computer" Vergouw et al., 2020

"No...I cannot... I am only able to use WhatsApp, receiving or making a call" Rochmawati et al., 2022

"So I got an iPhone, it's daunting as a 90-year-old. It's got a billion buttons. I went out and purchased the manual, which is not produced by Apple-it's produced by other people because Apple just presumes that people know how to use it [iPhone]" Mao et al., 2022

"But I really get furious when things do not work properly. That is difficult for me. I try but when it does not work I tend to give up" Vergouw et al., 2020

a desire to learn computer skills - Watt et al., 2022

"(Teleconsultations) That's no good, I barely know how to handle a cell phone. What are they talking to me about?" Loza et al., 2021

"I'm not computer literate" Watt et al., 2022

"It's probably not beyond my capabilities, but I just have not done it" Watt et al., 2022

Older adults emphasised the need and desire to possess adequate skills, knowledge and resources in order to utilise the online eHealth applications - Vergouw et al., 2020

"This is unclear, it is too difficult for me" Vergouw et al., 2020

some patients and caregivers voiced confidence in their ability to use a telephone, but not in their ability to use a smartphone or computer - Watt et al., 2022

fear of making mistakes or the uncertainty about a message being delivered to the right recipient - Vergouw et al., 2020

"I don't know how to upload them onto my (cloud drive)... I mean I know how to send it to myself with an email or text, but I don't know how to directly upload it and I don't know... even really know if those are different things" Johnson et al., 2021

"I think it's too late for me to start to learn things, you know" Watt et al., 2022

"Given our age and not being computer literate, I think we nailed it" Iyer et al., 2021

Participants mostly preferred using the computer or laptop instead of a tablet due to the screen size - Vergouw et al., 2020

participants showed limited health technology skills - only able to use their smartphone for WhatsApp - Rochmawati et al., 2022

Online Security

"I have this feeling like the laptop computer is more secure than my smart phone is, but for an app what we're talking about here for the BCC...using a smart phone for that, I don't think I'd have a problem with that. I get concerned over the financial sort of stuff... So I know there's a certain amount of security and I'm just not used to it" Johnson et al., 2021

"Cause I don't feel that safe now (...) [like I did] when the nurses at the healthcare centre were checking. (...) [the remote patient monitoring system] has got so big now so that they have needed to hire and they have focused more on nurses' digital [competence] instead of hiring a nurse who is personal and then teaching [that Registered Nurse] the digital. (...). So now they've got this one [Registered Nurse] that's really good at computers and knows all about phones, tablets, and stuff. I think they have made a mistake" Lindberg et al., 2022

Concerned about the security of the mHealth devices - Johnson et al., 2021

risk of privacy leakage will decrease the perceived usefulness of mHealth services - Pan et al., 2021

Concerns over medical information being shared - Vergouw et al., 2020

Not feeling safe as lots of people can access their patient monitoring system - Lindberg et al., 2021

Not trusting it - risk of privacy. Worried about the security of their personal data - Pan et al., 2021

Access to digital devices and the internet

Access to computers and the internet - Watt et al., 2022

"I have a really old device..." Mao et al., 2022

Not having enough space on their device - Pan et al., 2021

Accessibility to health technology - Rochmawati et al., 2022

All participants had access to technology for contact with health care, but access to the technology was not equal to successful use of it - Jakobsson et al., 2019

Low internet speed - Pan et al., 2021

participants expressed their needs to be better informed about the availability of the applications either by letter, information sheets, advertisements in the local newspaper or email - Vergouw et al., 2020

unfamiliarity with he applications - Vergouw et al., 2020

a need for older adults to have access to technology and opportunities to learn how to use it - Watt et al., 2022

Lack of awareness

some people were not aware of ehealth services - Jakobsson et al., 2019

limited understanding of the applications (comprehension) and how to enter the right information (data entry) as major impediments - Vergouw et al., 2020

lack of familiarity with technology and telemedicine platform - Mao et al., 2022

"He wasn't a snooty doctor, but he was like any friend, actually. So, he was very easy to talk with" Jakobsson et al., 2019

Registered Nurses' practical support had been essential in enabling them to be able to use digital applications - Lindberg et al., 2021

"But because we... have been meeting with the doctors many times... we know quite a while already... so it's OK, but if for a new patient and they'll meet the doctor, I think it's good face-to-face" Watt et al., 2022

perceived impacts on the patient-provider relationship has the potential to be both a barrier and facilitator to mHealth use. These influences attitudes and beliefs - Johnson et al., 2021

Relationship with healthcare provider(s)

"It was a bit weird, I think. (...) [The Registered Nurse] asked if I wanted her to be there and I thought that it would feel good if she was" Lindberg et al., 2021

People didn't feel safe after his relationship with local primary healthcare has been geared more towards digital support and resulted in fewer personal interactions - Lindberg et al., 2021

Developing a personal contact with the health care provider included being seen, getting help and being taken care of, which created a good and genuine feeling - Jakobsson et al., 2019

"What attracted me to this was this whole thing with, what do you call it, 'preventive [digital] care'. They [the local Registered Nurse] told me about it and that maybe in the future it'll be further developed" Lindberg et al., 2021

importance of interpersonal relationships with healthcare providers - Jakobsson et al., 2019

the importance of a pre-existing doctor-patient-caregiver relationship and their presence to provide collateral history and facilitate aspects of the assessment as key facilitators of virtual care effectiveness - Watt et al., 2022

In person preference

"Through video, there is no way to measure blood pressure...I can only tell you I don't feel comfortable" Mao et al., 2022

The doctor said if we meet in person it will have a different effect... It is different if we communicate with inanimate objects. It is said that it is healthier to meet face-to-face, you know, but I am not sure if that is correct (smiles)... I just heard from smart people" Rochmawati et al., 2022

"If you have it [the appointment time] on your mobile, it will sort of disappear as things do on your mobile. Here, on the refrigerator one can see the to-do-notes" Jakobsson et al., 2019

"I would rather that the doctor can actually touch me, examine me with a stethoscope, or see if a part is tender...I also think in-person communication is sometimes better..." Mao et al., 2022

"A friend of mine made one of these consultations; I asked her: – and what did the doctor do to you? – No, nothing, he asked me how I was doing. And that is useless... You have to get checked, if they can't physically check you, it's not medicine" Loza et al., 2021

"I feel more comfortable to talk with a physician face to face" Pan et al., 2021

caregivers worried that medical conditions such as hypertension and weight loss were not being identified because routine components of in-person care were not being provided. - Watt et al., 2022

the desire of personal contact and seeing peoples expressions - Mao et al., 2022

"I like seeing people's eyes" Pan et al., 2021

"I'm not a health professional, I prefer to see a physician" Pan et al., 2021

"I think that really matters with older people because... I think [they] need a more detailed kind of assessment. And I just don't know how that's possible with a virtual meeting" Watt et al., 2022

"I told you, you know, talk with people in person is much, much better" Watt et al., 2022

Speaking to a human is better than technology - Mao et al., 2022

"I would rather make a phone call to arrange an appointment and prefer to talk face-to-face to the physician" Vergouw et al., 2020

preference to personally visit their general practice instead of using the applications due to the closeness of their home to the practice itself - Vergouw et al., 2020

Most interviewees believed that in-person assessments were more accurate than virtual assessments - Watt et al., 2022

"Physicians cannot see and feel how I am web-based" Pan et al., 2021

"ohhhh, I have never tried the online one. If the online is implemented, I prefer to come to the clinic because my home is very close to the clinic" Rochmawati et al., 2022

"If I have limitations in daily functioning and I am alone, I think I will have no need for applications. Then I prefer to make a phone call, to have more personal contact" Vergouw et al., 2020

Preferred personal contact - offered reassurance and the ability to ask questions right away - Vergouw et al., 2020

some of the people interviewed stated that consultation via telemedicine was not always sufficient, as they consider the physical examination a crucial tool for the understanding and diagnosis for which they consult - Loza et al., 2021

"The nurse has toddlers, so of course she'd want to work more from home, this is perfect for her, as she can sit by the computer and still help us" Lindberg et al., 2021

"Yes, the advantage is that I can go on my device at my own leisure in my own time, without being limited to the allotted 10 minutes of the physician time. At my leisure I can review previous results and information" Vergouw et al., 2020

"I sent an email to the doctor and she sent me the prescription, I had no problems (...) The medication is sent to me by WhatsApp by the Dr., I show up with the card at the pharmacy and they give it to me without any problem. I go once a month" Loza et al., 2021

"I believe that contact with the general practitioner will be strengthened by using the applications more frequently even though you can't see her. Thus, easy to get in contact with the GP and get a proper assessment on my current health status" Vergouw et al., 2020

Convenience

convenience and efficiency of the applications. Participants stated that they could use the applications anywhere, any time and at their own convenience - Vergouw et al., 2020

a high degree of satisfaction over the time efficiency, travel time saved, and cost savings of virtual visits - Iyer et al., 2021

"I would do it [video visit] more often than not. I think you've done a great job. It's a godsend" Iyer et al., 2021

"Every 15 days video call with the doctor to check up on me, in case my blood pressure suddenly drops" Loza et al., 2021

online eHealth applications are an easy way to get and stay in contact with the GP - Vergouw et al., 2020

the device's ease of use, convenience, and attractive design make technology appealing for the older people - Rochmawati et al., 2022
