# Supplementary material for: What are the determinants of older people adopting communicative e-health services: a meta-ethnography
Source: BMC Health Serv Res. 2024 Jan 11;24:60. doi: 10.1186/s12913-023-10372-3 (PMC10785477; doi:10.1186/s12913-023-10372-3)
Supplement: Supplementary file 2 — Additional file 2. A list of first and second order constructs. [file 12913_2023_10372_MOESM2_ESM.pdf]

	First Order Construct	Second Order Construct
Health Barriers	"Only thing is that I don't have the dexterity… ((42) p. 9)	challenging for older adults to access virtual care, including hearing impairment, cognitive impairment and need for translation services ((44) p. 4)<br>Complexities in caring for older adults such as the presence of multiple chronic conditions… make virtual care challenging ((44) p. 3)<br>For patients with hearing impairment, providers reported challenges during telephone visits, but not video visits ((39) p. 5)<br>Impaired motor skills and difficulty with the touch functioning ((38) p. 5)
Support Networks	"If it seemed too complicated to do, I mean there are enough people around… There are two people in the family that are in IT and are extremely savvy and could help and can teach and can support me" ((42) p. 6)	some of the participants stated that they felt resistant to asking for support ((38) p. 6)
	"I was here [at Healthcare Centre 1] and one of the staff showed me and connected it so that they could synchronise it with my phone. You had to download some 'app'. (...) I cannot do that. She [a local Registered Nurse] did that" ((32) p. 5)	Lack of support was seen as a barrier to learning how to use mHealth apps ((40) p. 4)
	"I do not browse by myself, usually my children will do the search if I experience symptoms or health complaints then they will send the information to me" ((47) p. 5)	Openness to technology can be persuaded by caregivers. The availability of support for older people is important in health technology adoption among older people ((45) p. 6)
	"As long as my kids and my wife are alive, it would be okay… Because I'm not a techy guy…So I'd be okay with it if I've got somebody to help me" ((42) p. 6)	They also highlight family support, as younger family members have better use of these technologies ((43) p. 14)
	"I need a person to sit down with me next to my computer to help me set up my account: here's the icon you click on, the name of your account, where you keep your password, how you enter and use it...I need personal help" ((43) p. 6)	Some participants expressed worries about not being able to maintain their health care contacts without the support ((38) p. 6)
	"If I use English, it will be very hard. I am comfortable with computers, and am willing to give it a try...I hope there is someone who speaks Chinese to help with technology" ((43) p. 6)	Having a caregiver present to facilitate virtual care was identified as a key facilitator of videoconference-based virtual care for patients ((44) p. 5)
	"[The telemedicine platform is] very complicated – much more so than Zoom. I have very poor vision and I'm old and it's no	All participants stated that the applications have to be designed clearly and simply ((42) p. 7)

Application interface and design	good for me... I think just having help at the time I have to get on is the best or you should switch to a simpler system” ((43) p. 6)	
	“There are many steps to book the [telemedicine] app, I have received a lot of information (e.g., Email) on how to connect. I feel like I am not smart enough to persist through the whole [set of instructions]” ((43) p. 5)	the design of app interface will directly affect the perceived ease of use ((13) p. 14)
	“Yes, how to operate the applications was all clearly indicated, there was nothing wrong there” ((44) p. 9)	Some noted that they are familiar with platforms they already use such as Zoom or WeChat and would prefer if their care providers switched to simpler platforms for telemedicine video visits ((41) p. 6)
	“Accessibility is important to me. You should be easily able to log on using your username and password” ((44) p. 7)	difficulty with the log in and too many screens making things complex ((42) p. 6)
Digital Literacy	“(Teleconsultations) That’s no good, I barely know how to handle a cell phone. What are they talking to me about?” ((45) p. 18)	some patients and caregivers voiced confidence in their ability to use a telephone, but not in their ability to use a smartphone or computer ((44) p. 5)
	“I don’t know how to upload them onto my (cloud drive)… I mean I know how to send it to myself with an email or text, but I don’t know how to directly upload it and I don’t know… even really know if those are different things” ((42) p. 6)	Most of the participants showed limited health technology skills – only able to use their smartphone for WhatsApp ((45) p. 6)
	“But we did not grow up with the computer. I would rather make a phone call to arrange an appointment” ((44) p. 5)	fear of making mistakes or the uncertainty about a message being delivered to the right recipient ((42) p. 5)
	“I think it’s too late for me to start to learn things, you know” ((46) p. 4)	Older adults emphasised the need and desire to possess adequate skills, knowledge and resources in order to utilise the online eHealth applications ((42) p. 3)
	“Given our age and not being computer literate, I think we nailed it” ((41) p. 6)	
	“It’s probably not beyond my capabilities, but I just have not done it” ((46) p. 5)	
	“No…I cannot… I am only able to use WhatsApp, receiving or making a call” ((47) p. 5)	
	“I got an iPhone, it’s daunting as a 90-year-old. It’s got a billion buttons. I went out and purchased the manual, which is not produced by Apple-it’s produced by other people because Apple just presumes that people know how to use it [iPhone]” ((43) p. 5)	
	“I have this feeling like the laptop computer is more secure than my smart phone is” ((42) p. 6)	concern about what might happen to their private medical history information while using these eHealth applications. ((42) p. 6)

Online Security	"Cause I don't feel that safe now (...) [like I did] when the nurses at the healthcare centre were checking. (...) [the remote patient monitoring system] has got so big now so that they have needed to hire and they have focused more on nurses' digital [competence] instead of hiring a nurse who is personal and then teaching [that Registered Nurse] the digital. (...). So now they've got this one [Registered Nurse] that's really good at computers and knows all about phones, tablets, and stuff. I think they have made a mistake" ((32) p. 4)	risk of privacy leakage will decrease the perceived usefulness of mHealth services ((13) p. 14)<br>When the app asked for access to their location or photos, they gave up, worrying about the security of their personal data ((13) p. 12)
Access to Digital Devices and the Internet	"I have a really old device..." ((43) p. 5)	one participant abandoned the tests when downloading a new app; there was not enough storage space in her phone ((13) p. 12)<br>Accessibility to health technology was limited ((45) p. 6)<br>All participants had access to technology for contact with health care, but access to the technology was not equal to successful use of It ((38) p. 5)<br>Most importantly, patients or caregivers without a computer or smartphone (and adequate sound and video equipment) or reliable internet access would not be able to access videoconference based virtual care ((44) p. 5)<br>Long waiting for system responses often frustrates people when the responses come slowly owing to the unstable connection of the internet or low speed ((13) p. 14)
Lack of Awareness		participants expressed their needs to be better informed about the availability of the applications either by letter, information sheets, advertisements in the local newspaper or email ((42) p. 5)<br>Patients, caregivers and healthcare providers expressed a need for older adults to have access to technology and opportunities to learn how to use it ((44) p. 4)<br>Many of the participants in the present study were not aware of eHealth services ((38) p. 7)<br>The challenges of adopting telemedicine for older adults is partly due to a lack of familiarity with video and internet technology ((41) p. 6)<br>unfamiliarity with he applications ((42) p. 5)<br>limited understanding of the applications (comprehension) and how to enter the right information (data entry) as major impediments ((42) p. 6)

Relationship with Healthcare Providers	“He wasn’t a snooty doctor, but he was like any friend, actually. So, he was very easy to talk with” ((38) p. 6)<br>“But because we... have been meeting with the doctors many times... we know quite a while already... so it’s OK, but if for a new patient and they’ll meet the doctor, I think it’s good face-to-face” ((46) p. 4)<br>“It was a bit weird, I think, (…) (The Registered Nurse) asked if I wanted her to be there and I thought that it would feel good if she was” ((32) p. 4)	importance of interpersonal relationships with healthcare providers ((38) p. 8)<br><br>It is novel in its integration of the patient- and provider-level factor that influences attitudes and beliefs regarding the domains of mHealth and active surveillance ((40) p. 7)<br><br>One man said that he did not feel safe after his relationship with local primary health care had been geared more towards digital support, and included fewer personal interactions ((32) p. 4)<br>the importance of a pre-existing doctor patient caregiver relationship and their presence to provide collateral history and facilitate aspects of the assessment as key facilitators of virtual care effectiveness ((44) p. 4)
In Person Preference	“Physicians cannot see and feel how I am web-based” (13) p. 10)<br><br>“Through video, there is no way to measure blood pressure...I can only tell you I don’t feel comfortable” ((43) p. 5)<br><br>“I feel more comfortable to talk with a physician face to face” ((13) p. 10)<br><br>"I like seeing people’s eyes" ((13) p. 10)<br><br>"If I have limitations in daily functioning and I am alone, I think I will have no need for applications. Then I prefer to make a phone call, to have more personal contact" ((42) p. 5)<br><br>“I have never tried the online one. If the online is implemented, I prefer to come to the clinic because my home is very close to the clinic” ((47) p. 6)<br>“I would rather make a phone call to arrange an appointment and prefer to talk face-to-face to the physician” ((43) p. 5)<br>“A friend of mine made one of these consultations; I asked her: – and what did the doctor do to you? – No, nothing, he asked me how I was doing. And that is useless... You have to get checked, if they can’t physically check you, it’s not medicine” ((45) p. 18)	Most interviewees believed that in-person assessments were more accurate than virtual assessments ((44) p. 4)<br>preferring to speak to a human rather than technology and the experience of desiring personal contact and seeing expressions ((41) p. 5)<br>Other participants describe preferring to speak to a human rather than technology and the experience of desiring personal contact and seeing expressions ((41) p. 5)<br>Preferred personal contact - offered reassurance and the ability to ask questions right away ((42) p. 5)<br>some of the people interviewed stated that consultation via telemedicine was not always sufficient, as they consider the physical examination a crucial tool for the understanding and diagnosis for which they consult ((43) p. 18)<br>caregivers worried that medical conditions such as hypertension and weight loss were not being identified because routine components of in person care were not being provided. ((44) p. 4)

	“I would rather that the doctor can actually touch me, examine me with a stethoscope, or see if a part is tender...I also think in-person communication is sometimes better...” ((43) p. 5)	
Convenience	“I sent an email to the doctor and she sent me the prescription, I had no problems (...) The medication is sent to me by WhatsApp by the Dr., I show up with the card at the pharmacy and they give it to me without any problem. I go once a month” ((45) p. 14)	online eHealth applications are an easy way to get and stay in contact with the GP ((42) p. 10)
	“Yes, the advantage is that I can go on my device at my own leisure in my own time, without being limited to the allotted 10 minutes of the physician time. At my leisure I can review previous results and information” ((44) p. 6)	a high degree of satisfaction over the time efficiency, travel time saved, and cost savings of virtual visits ((39) p. 7)
	“The nurse has toddlers, so of course she’d want to work more from home, this is perfect for her, as she can sit by the computer and still help us” ((32) p. 6)	the device's ease of use, convenience, and attractive design make technology appealing for the older people ((45) p. 7)
	“Every 15 days video call with the doctor to check up on me, in case my blood pressure suddenly drops” ((45) p. 12)	convenience and efficiency of the applications. Participants stated that they could use the applications anywhere, any time and at their own convenience ((42) p. 6)
	“I believe that contact with the general practitioner will be strengthened by using the applications more frequently even though you can’t see her. Thus, easy to get in contact with the GP and get a proper assessment on my current health status” ((44) p. 7)	
