# Supplementary material for: What are the determinants of older people adopting communicative e-health services: a meta-ethnography
Source: BMC Health Serv Res. 2024 Jan 11;24:60. doi: 10.1186/s12913-023-10372-3 (PMC10785477; doi:10.1186/s12913-023-10372-3)
Supplement: Supplementary file 3 — Additional file 3: Table 5. Summary table with additional information. [file 12913_2023_10372_MOESM3_ESM.pdf]

Table 5 Summary table with additional information

Author & Year	Title	Journal	DOI	Study Outcomes	Key Conclusions
**Jakobsson et al., 2019**	Experiences from using eHealth in contact with health care among older adults with cognitive impairment	Scandinavian Journal of Caring Sciences	10.1111/scs.12634	The participants' use of eHealth was influenced by a wide range of aspects; for example, awareness of eHealth; limitations from cognitive impairments; uncertainty/ certainty to one's own abilities regarding technology use and the value of interpersonal relationships, all united by habits. The findings also show that the older adults experienced several barriers in using the technology in contact with health care which also affected their use of eHealth	There is a need to develop interventions which can reach older adults. Replacing personal services with technology is not a solution which suits everyone.
**Vergouw et al., 2020**	Needs, barriers and facilitators of older adults towards eHealth in general practice: A qualitative study	Primary Health Care Research & Development	10.1017/ S1463423620000547	It was observed that personal contact is an important need for feeling reassured. That non-familiarity with online services and mismatch with health care needs turned out as barriers. And the ability with quick access to results along with more possibilities for easy contact with the GP facilitates in using the eHealth applications	A user-friendly interface supports older adults in using online eHealth applications. Attention is needed to invite older adults in the development process of new applications to ensure its feasibility and to ensure that their needs are fulfilled
**Lindberg et al., 2021**	Older people and rural eHealth: Perceptions of caring relations and their effects on engagement in digital primary health care	Scandinavian Journal of Caring Sciences	10.1111/scs.12953	In the study, participants often welcomed eHealth into their lives. 'Younger old' participants for example, tended to be relatively accepting towards digital technology. Still, most participants were sceptical and feared digital health care would be at the expense of local inperson relations. Noticeably, the study participants had diverse opinions about eHealth but still chose to engage in digital health care	Results provide insights into matters of quality, access, and equality in rural primary health care, specifically in relation to older people.

**Iyer et al., 2021**	Converting a geriatrics clinic to virtual visits during COVID-19: A case study	Journal of Primary Care & Community Health	10.1177/21501327211000235	Results showed that telemedicine care via phone or video is not only feasible for geriatric primary care services, but also accepted and appreciated in this very frail, older adult clinic. There was a large time investment in helping this population set up the technology and software for video visits, but this worthwhile investment resulted in high patient and provider satisfaction.	Telemedicine during a pandemic comes with challenges, including the time needed to prepare and assist patients with technology, securing the availability of devices, and ensuring sufficient bandwidth to support surge usage. Despite these challenges, satisfaction amongst older patients, caregivers and providers with virtual visits was generally high. Providers completed the majority of clinical care virtually, and patients expressed interest in continuing with telemedicine after the pandemic
**Johnson et al., 2021**	Barriers and facilitators to mobile health and active surveillance use among older adults with skin disease	Health Expectations	10.1111/hex.13229	The study revealed several key factors shaping patients' willingness to use active surveillance using mHealth for dermatologic disease and low-risk skin cancers. When determining whether older adults would use mHealth, it is important to consider the usability of the app, changes to the patient-provider interaction, whether the technology aligns with a person's values and the presence or absence of support systems such as caregivers	Factors influencing patients acceptance and adoption of active surveillance in deermatology is similar to those other specialties

**Loza et al., 2021**	A qualitative study on the elderly and accessibility to health services during the COVID-19 lockdown in Buenos Aires, Argentina - Part 2	Medwave	10.5867/medwave.2021.04.8192	The suspension of face-to-face consultations, either due to the closure of healthcare centers or to changes in their services (providing only services on spontaneous demand or on-call), gave rise to the emergence of information and communication technologies. Although some technologies dominated, predominantly WhatsApp, most of the participants preferred face-to-face consultation, often pointing to barriers to its use. In this way, the need for digital literacy of older adults to reduce the inequity generated by the digital divide becomes present in this work.	The COVID-19 pandemic generated a new scenario in which accessibility to the health system was affected at the expense of reduced access to face-to-face consultations. The emerging needs forced the development of new care strategies, most of which focused on information and communication technologies. Although this provided a solution for many older adults, it also generated new exclusions due to preexisting technological gaps.
**Pan et al., 2021**	Perception and initial adoption of mobile health services of older adults in London: Mixed methods investigation	JMIR Ageing	10.2196/30420	Findings show that the lack of obvious advantage, low reliability, scary information, and risk of privacy leakage will decrease the perceived usefulness of mHealth services; the design of app interface will directly affect the perceived ease of use; aging factors, especially the generation gap, will make mHealth difficult for older adults to used, and identified the barriers that older adults face during their initial adoption of mHealth apps. Access to technology, the way of interaction, the risk of money loss, heavy workload to use an mHealth app, and the different lifestyles of older adults have a great influence on older adults' adoption of mHealth services.	The perceptions of mHealth services of older adults were investigated; the barriers that older adults may face in the initial adoption stage were identified. On the basis of the synthesis of these results, design suggestions were proposed, including technical improvement, free trial, information clarification, and participatory design. They will help inform the design of mHealth services to benefit older adults.

**Watt et al., 2022**	Barriers and facilitators to virtual care in a geriatric medicine clinic: A semi-structured interview study of patient, caregiver and healthcare provider perspectives	Age and Ageing	10.1093/ageing/afab218	The results identified barriers and facilitators to virtual care implementation that were related to the COVID-19 pandemic and other barriers and facilitators related to the added complexity of caring for older adults. Uncertain accuracy of virtual care, inequity in access to virtual care, and the importance of caring for the patient-caregiver dyad, among others, will continue to be important even after the COVID19 pandemic. Patient, caregiver, and healthcare provider knowledge, skills, belief in capabilities, and environmental context and resources will be key domains to target in future studies of virtual care implementation.	In conclusion, although patients, caregivers and healthcare providers voiced support for continuing virtual care after the COVID-19 pandemic, there were caveats. More research is needed to understand how virtual assessments can complement in-person assessments. Moreover, the implementation and sustainability of virtual care for older adults attending geriatric medicine clinics will depend on greater integration of technology into the lives of older adults and clinics
**Rochmawati et al., 2022**	Acceptance of e-health technology among older people: A qualitative study	Nursing & Health Sciences	10.1111/nhs.12939	Most of the participants showed enthusiasm for ehealth monitoring. A participant used a smartwatch for activities/ sports; however, no one had used mobile phone health apps. The participants stated that they had little knowledge about digital literacy and limitations in the use of digital tools and were not familiar with the use of health technology	Older people are willing to use technology to seek health information online. The participants in our study still preferred face-to-face interactions with healthcare professionals. Althoug some older people acknowledged the importance of home health technology, they had skills limitations in terms of being able to use devices. The findings of our study emphasize the need to develop targeted solutions for older people with different technology capabilities

**Mao et al., 2022**	Barriers to telemedicine video visits for older adults in independent living facilities: Mixed methods cross-sectional needs assessment	JMIR Ageing	e34326	Participants identified several barriers regarding telemedicine use, especially in conducting video visits. The top barriers included not knowing how to connect to the platform (including language barriers that make instructions difficult to understand), not being familiar with the technology, difficulty hearing, and lack of interest in seeing providers outside of the clinic.	Older adults make up many patients in our health care system, though their perspectives are rarely formally elicited. Decreased use of telemedicine exposes this already vulnerable population to further health care inequities. Our study will not only inform our own quality improvement initiatives in our community but also, we hope, open the door to larger scale studies in understanding the patient experience as telemedicine becomes a larger cornerstone of care delivery.
